# Effect of Er,Cr:YSGG Laser at Different Output Powers on the Micromorphology and the Bond Property of Non-Carious Sclerotic Dentin to Resin Composites

**DOI:** 10.1371/journal.pone.0142311

**Published:** 2015-11-06

**Authors:** Xiang Sun, Jinghao Ban, Xinjia Sha, Weiguo Wang, Yang Jiao, Wanshan Wang, Yanwei Yang, Jingjing Wei, Lijuan Shen, Jihua Chen

**Affiliations:** 1 State Key Laboratory of Military Stomatology, Department of VIP Dental Care, School of Stomatology, The Fourth Military Medical University, Xi'an, P.R. China; 2 State Key Laboratory of Military Stomatology, Department of Preventive Dentistry, School of Stomatology, The Fourth Military Medical University, Xi'an, P.R. China; 3 Department of Stomatology, No.117 Hospital of PLA, Hangzhou, P.R. China; 4 State Key Laboratory of Military Stomatology, Department of Prosthodontics, School of Stomatology, The Fourth Military Medical University, Xi'an, P.R. China; 5 Shaanxi Key Laboratory of Stomatology, Xi'an, P.R. China; 6 State Key Laboratory of Military Stomatology, Department of Dental Materials, School of Stomatology, The Fourth Military Medical University, Xi'an, P.R. China; 7 Department of Stomatology, Lanzhou General Hospital Lanzhou Command of PLA, Lanzhou, China; University of Brescia, ITALY

## Abstract

**Background:**

The objective of this study was to investigate the influence of Er,Cr:YSGG laser irradiated at different powers on the micromorphology and the bonding property of non-carious sclerotic dentin to resin composites.

**Methods:**

Two hundred bovine incisors characterized by non-carious sclerotic dentin were selected, and the seventy-two teeth of which for surface morphological analysis were divided into nine groups according to various treatments (A: the control group, B: only treated with the adhesive Adper Easy One, C: diamond bur polishing followed by Adper Easy One, D-I: Er,Cr:YSGG laser irradiating at 1W, 2W, 3W, 4W, 5W, 6W output power, respectively, followed by Adper Easy One). The surface roughness values were measured by the non-contact three-dimensional morphology scanner, then the surface micromorphologies of surfaces in all groups were assessed by scanning electron microscopy (SEM); meanwhile, Image Pro-Plus 6.0 software was used to measure the relative percentage of open tubules on SEM images. The rest, one hundred twenty-eight teeth for bond strength test, were divided into eight groups according to the different treatments (A: only treated with the adhesive Adper Easy One, B: diamond bur polishing followed by the above adhesive, C-H: Er,Cr:YSGG laser irradiating at 1 W, 2 W, 3 W, 4 W, 5 W, 6 W output power, respectively, followed by the above adhesive), and each group was subsequently divided into two subgroups according to whether aging is performed (immediately tested and after thermocycling). Micro-shear bond strength test was used to evaluate the bond strength.

**Results:**

The 4W laser group showed the highest roughness value (30.84±1.93μm), which was statistically higher than the control group and the diamond bur groups (p<0.05). The mean percentages ((27.8±1.8)%, (28.0±2.2)%, (30.0±1.9)%) of open tubules area in the 4W, 5W, 6W group were higher than other groups (p<0.05). The 4W laser group showed the highest micro-shear bond strength not only in immediately tested (17.60±2.55 PMa) but after thermocycling (14.35±2.08MPa).

**Conclusion:**

The Er,Cr:YSGG laser at 4W power can effectively improve the bonding property between non-carious sclerotic dentin and resin composites by increasing the roughness and mean percentage area of open tubules.

## Introduction

Due to the complexity of dentin, bonding of dental materials to dentin substrates has attracted increasing attention among dental researchers. Although the bonding effectiveness of most adhesive systems is shown to be favorable in most studies, the results are commonly based on binding to sound dentin. However, a variety of pathological dentin substrates are also encountered in clinical practice, which mainly includes carious and non-carious sclerotic dentin. Irrespective of the bonding strategy used, bonding to pathologically altered substrates, especially non-carious sclerotic dentin, is typically compromised [1]. In vitro studies [2] have demonstrated that for etch-and-rinse adhesives, bond strength values for sclerotic dentin are 25%–40% lower than those achieved for sound dentin. This reduction is due to partial or complete obliteration of the dentinal tubules with mineral crystals and the presence of an acid-resistant hyper-mineralized layer that acts as an acid-resistant substrate to hinder penetration of the resin. Furthermore, due to the deposition of mineral crystallites on non-carious sclerotic dentin, dentin collagen fibers encounter mineralized degeneration, which results in the replacement of collagen-rich intertubular dentin with highly mineralized peritubular dentin [3–5].

Previous studies have shown that roughening the surface can markedly increase the bonding area between the substrate and resin, which may improve bonding strength [6, 7]. Therefore, it has been proposed that an effective method for treating pathological dentin substrates, which could increase surface roughness as well as partially or completely remove mineral crystals in dentinal tubules to improve the formation of resin tags, can lead to improve the bond of non-carious sclerotic dentin to resin composites. Previous studies have suggested that bonding to human sclerotic dentin can be improved by changing the adhesive protocol, such as pretreatment with diamond bur polishing [8,9], doubling the conditioning time of self-etching adhesives [1], and pretreatment with phosphoric acid [9, 10], etc. However, the effectiveness of these approaches has been insufficient for clinical application [1, 11, 12].

Recently, the technological developments have resulted in more widespread application of lasers in the field of oral health care [13–15]. The lasers used most often in dental applications include Nd:YAG, Er:YAG, and Er,Cr:YSGG lasers, and so on. Especially, Er,Cr:YSGG laser, as a new hydrokinetic biological laser system, can effectively remove dental hard tissue without inducing the formation of a smear layer or causing heat injury on the tooth surface or to pulp, respectively [16, 17]. Specific advantages of the Er,Cr:YSGG laser have led to its application in treating dental hard tissues. For enamel, Er,Cr:YSGG laser treatment offers a more effective alternative to conventional acid-etching [18, 19]. With respect to dentin, studies [20, 21] have shown lower bond strengths compared to those achieved after conventional diamond bur treatment due to the effect of the Er,Cr:YSGG laser on the collagen fibers.

Many factors are known to influence the effects of Er,Cr:YSGG laser treatment on dental hard tissues, such as the water/air ratio, the repetition rate, and the output power. Some studies [22, 23] have specifically investigated the influence of the water/air ratio and the repetition rate for Er,Cr:YSGG laser treatment on the bond strength of dental hard tissues. Unfortunately, to date there has been a lack of studies investigating the influence of Er,Cr:YSGG laser treatment at different output powers on bonding to non-carious sclerotic dentin. Therefore, the aim of this study was to evaluate the influence of Er,Cr:YSGG laser treatment at various output powers on the bonding strength of resin to non-carious sclerotic dentin and then to determine the optimal power of the Er,Cr:YSGG laser for clinical usage. The null hypothesis tested was that different treatments had no effect on the micromorphology and micro-shear bond strength of non-carious sclerotic dentin to resin composites.

## Materials and Methods

### 1. Teeth collection

Two hundred freshly extracted bovine incisors with exposed non-carious sclerotic dentin on the lingual surface, clinically characterized by a vitreous appearance [24], physiologically discolored, caries-free, and undamaged [25] were collected from animals at least 3 years old [26] which had been slaughtered in Yunfeng Animal Husbandry, a local slaughterhouse located in the suburb of Xi’an, Shaanxi Province in China, for meat business. Non-carious sclerotic dentin was identified according to the North Carolina dentin sclerosis scale (Table 1) [27]. The sclerotic dentins located in the bovines we collected for the present study should be classified as Category 3 or 4 of the above sclerosis scale criteria by visual sense [25]. All teeth were stored in distilled water at 4°C for no more than 1 week before being used in experiments.

**Table 1:** North Carolina dentin sclerosis scale.

Category	Criteria
**1**	No sclerosis present; Dentin is light yellow or whitish in color with little discoloration; Dentin is opaque, with little translucency or transparency
**2**	More than category 1 but <50% between categories 1 and 4
**3**	More than category 1 but >50% between categories 1 and 4
**4**	Significant sclerosis present; Dentin is dark yellow or even discolored (brownish); Dentin has glassy appearance, with significant translucency or transparency

Based on the scale developed by Dr. Steven E. Duke of the University of Texas Health Science Center at San Antonio and modified by the Department of Operative Dentistry at the University of North Carolina School of Dentistry [29].

### 2. Surface morphological analysis

#### 2.1 Sample preparation

Seventy-two teeth were sectioned perpendicularly along the boundary of the sclerotic regions on the lingual surface with a water-cooled, low-speed diamond saw (SYJ-150, MTI Corp, Shenyang, China) and then sectioned along the labial surface to obtain cubic samples 3–4 mm in height, with sclerotic dentin exposed on the face of each sample. Next, all the specimens were randomly divided into nine groups of 8 teeth each, according to the main factor “surface treatment” (A: control group is untreated; B: adhesive Adper Easy One (3M ESPE, USA); C: diamond bur (SF-13, MANI.INC, Japan) polishing followed by adhesive Adper Easy One, D-I: an Er,Cr:YSGG laser (Millennium 2, Bio lase Technologies Inc, San Clemente, CA, USA) at 1W,2W,3W,4W,5W,6W output power, respectively, followed by adhesive Adper Easy One).

To all samples in the nine groups described above, the Adper Easy One adhesive was applied according to the manufacturer’s instructions. However, it was not light-cured. Then, the resin monomers of the self-etching primer were removed by immediately immersing the specimens in acetone for 5 min followed by immersion in deionized water for 5 min. After this, the specimens were immersed in 96% ethanol for 5 min and again in deionized water for 5 min [1, 28].

#### 2.2 Laser treatment

The Er,Cr:YSGG laser had a 2.78-μm wavelength, a pulse duration of 140–200 μs, a fixed 20-Hz repetition rate, and an adjustable average output power over the range of 0–6 W. The equipment was cooled with an air/water spray that was used at 65% air pressure and 55% water pressure [29, 30]. Laser energy was delivered through a fiber-optic system to a sapphire tip terminal 750 μm in diameter. The tip was bathed in an adjustable air/water spray during cutting. The area of each specimen was lased imbricately twice with a uniformly slow speed at a 90-degree angle to the surface and at a distance of 1 mm [19].

#### 2.3 Surface roughness measurement

The effect of all treatments on the mean surface roughness (Sa) of the non-carious sclerotic dentin was examined with a non-contact three-dimensional (3D) morphology scanner (PS50, Nanovea Co., USA). Two areas were scanned on every sample. The non-carious sclerotic dentin surfaces were positioned parallel to the device stage, and the focus was adjusted. The device was used with 18-μm steps at a frequency of 100 Hz, and the measurement area was 2 × 2 mm. After scanning, the surface roughness was analyzed with the Nanovea 3D software (Nanovea Co., USA).

#### 2.4 Scanning electron microscopy

After surface roughness treatment, the same specimens were used to observe the surface morphology. First, all specimens were ultrasonically cleaned with distilled water for 30 min. And then Specimens were dehydrated in ascending grades of ethanol and subjected to freeze drying. Finally, the samples were sputter-coated with gold (Sputtering SCD050, BalTec, Balzers, Liechtenstein) and examined using a field emission scanning electron microscope (Hitachi S-4800) at 15 kV operated in the secondary electron mode.

For SEM analysis, three pictures were taken from every sample. Measurements were performed as follows: the total area of dentin tubules in each image was recorded using the Image-Pro Plus 6.0 software (Media Cybernetics, USA) by a blinded researcher. Then, in the same image, the open tubule area was delineated by the above software and summed, and the ratio of open tubule area to total area was calculated to give the relative percentage of open tubule area of each specimen. Three measurements were averaged per sample for statistical purposes [2, 24].

### 3. Micro-shear bond strength test

#### 3.1 Specimen preparation

One hundred and twenty-eight teeth were sectioned perpendicularly along the boundary of the sclerotic regions on the lingual surface with a water-cooled, low-speed diamond saw and then sectioned along the labial surface to obtain cubic samples 3–4 mm in height, with sclerotic dentin exposed on the face of each sample. Next, all the specimens were randomly divided into eight groups according to the main factor “surface treatment” (A: control group merely with adhesive Adper Easy One; B: diamond bur polishing followed by adhesive Adper Easy One, C-H: an Er,Cr:YSGG laser at 1W,2W,3W,4W,5W,6W output power, respectively, followed by adhesive Adper Easy One). Each group was subsequently divided into two subgroups according to whether ageing was performed: immediately tested and thermocycling.

The surfaces of specimens in eight groups were treated with the adhesive Adper Easy One for 20s, and air-thinned for 5s. Prior to light-curing of the bonding resin, an iris cut from micro-bore tygon-tubing (R-3603, Norton Performance Plastic, Cleveland, OH, USA) with an internal diameter of 0.75mm and a height of 0.5mm was mounted on the surface of non-carious sclerotic dentin to restrict the bonding area. And a resin composites (Filtek Z250; 3M ESPE, St. Paul, MN, USA) was then filled into the cylinder. In this manner, very small cylinders of resin, approximately 0.75mm in diameter and 0.5mm in height, were bonded on the surface at three locations for one specimen. The immediately-tested subgroups were stored at room temperature (23°C) for 1 h prior to removing the tygon tubing, and then stored in water at 37°C for 24h. The thermocycling subgroups were subjected to aging treatment, as described in Section 3.2. All the specimens were used for micro-shear bond strength test(N = 24).

#### 3.2 Thermocycling aging

The specimens in the thermocycling subgroups were subjected to a thermocycling for 5000 times in the static water flow with temperature control between 5°C and 55°C with a dwell time of 60s at each temperature. This procedure was performed in a customized thermocycling apparatus (Oral thermocycling apparatus, Measurement and Control Technology Research Institute, Xi’an, China)

#### 3.3 Micro-shear bond strength test

A universal testing machine (Model AGS-10KNG; Shimadzu, Tokyo, Japan) equipped with a customized fixture was used for micro-shear bond strength test. The specimens with resin cylinders were adhered to testing device with a cyanoacrylate adhesive, which in turn was placed in universal testing machine for micro-shear testing. A thin wire (diameter 0.2 mm) was looped around the resin composites cylinder, making contact through half its circumference and was gently held flush against the resin/non-carious sclerotic dentin interface. A shear force was applied to each specimen at a cross-head speed of 1 mm/min until failure occurred. The resin/dentin interface for test, the wire loop and the center of the load cell were aligned as straight as possible to ensure the desired orientation in the shear test force. The maximum load values (N) were recorded and the values of mean failure load were calculated for all subgroups.

### 4. Statistical analysis

All the values obtained from the same tooth were averaged, and a tooth was considered as a statistical unit of the present investigation for the above Section 2.3 and 2.4 [31, 32]. The data for surface roughness were evaluated by one-way analysis of variance (ANOVA) and Tamhane tests. The data for the relative percentage of open tubule area were assessed by one-way ANOVA and Tukey’s test. Two-way ANOVA and Tukey’s test were used to analyse the data from the micro-shear test to evaluate the effect of different treatments. The level of significance was preset at α = 0.05.

## Results

### 1. Morphological analysis

#### 1.1 Surface roughness of sclerotic dentin

**Fig 1:**
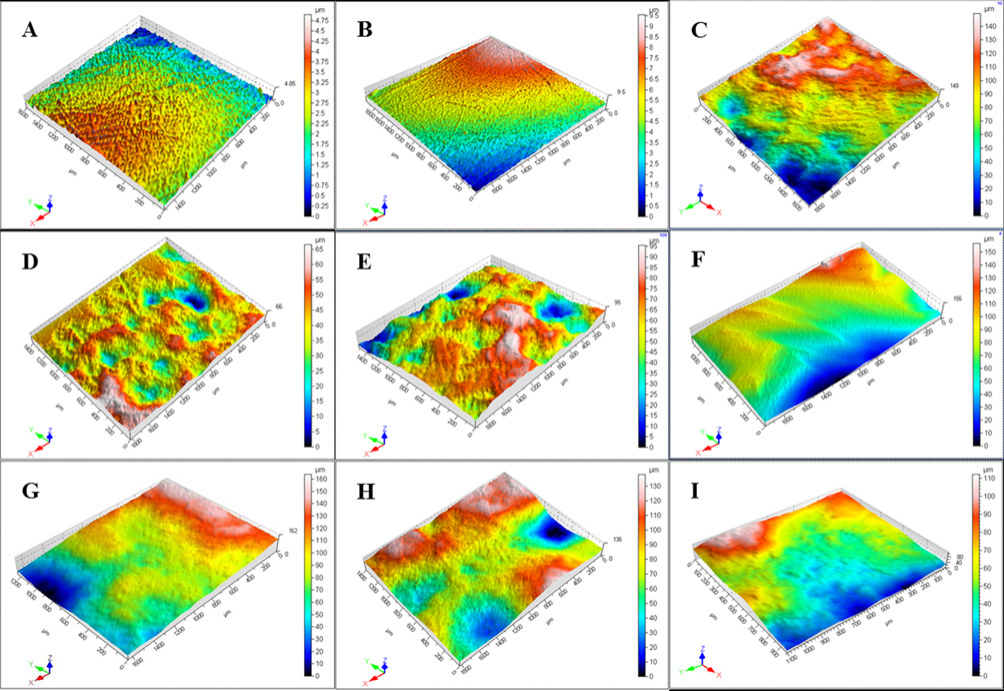
Representative 3D images of non-carious sclerotic dentin surfaces in all groups. (A) The control group without any treatment; (B) Adper Easy One adhesive treatment; (C) Diamond bur polishing followed by the application.of Adper Easy One adhesive; (D, E, F, G, H, and I) Er,Cr:YSGG laser irradiation at 1W, 2W, 3W, 4W, 5W, and 6 W, respectively, and followed by the application of Adper Easy One adhesive.

**Fig 2:**
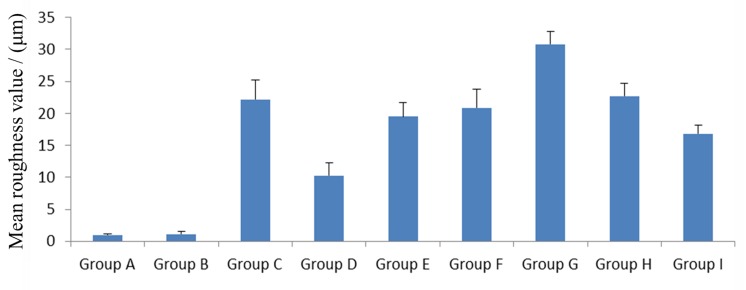
Mean surface roughness values of non-carious sclerotic dentin in all groups. (A) The control group without any treatment; (B) Adper Easy One adhesive treatment; (C) Diamond bur polishing followed by the application.of Adper Easy One adhesive; (D, E, F, G, H, and I) Er,Cr:YSGG laser irradiation at 1W, 2W, 3W, 4W, 5W, and 6 W, respectively, and followed by the application of Adper Easy One adhesive.

Fig 1 displays representative 3D surface morphology images obtained from all the groups with the 3D morphology scanner. The blue areas showed the lowest places while the pink areas were the highest places in the figures. The bigger the scaleplate maximum value on the right of the image was, the greater the difference between the lowest and highest place was, which means the higher roughness value. Meanwhile, the mean Sa values of non-carious sclerotic dentin, which corresponded to the Fig 1 images in all groups, could be obtained and were showed in Fig 2. By the statistical analysis, the 4W laser group showed the highest Sa value among the groups (p<0.05) while the control group had the lowest Sa value which was similar to the etching group (p = 1.0). The Sa value of the diamond bur group showed no significant difference with the 2W, 3W and 5W laser group (p>0.05), and was statistically higher than the control group, etching group, 1W and 6W group (p<0.05). In laser groups, the 5W laser group as well as the 6W laser group showed no significant difference with 2W and 3W laser group (p>0.05).

#### 1.2 Micromorphology of the surfaces of non-carious sclerotic dentin

**Fig 3:**
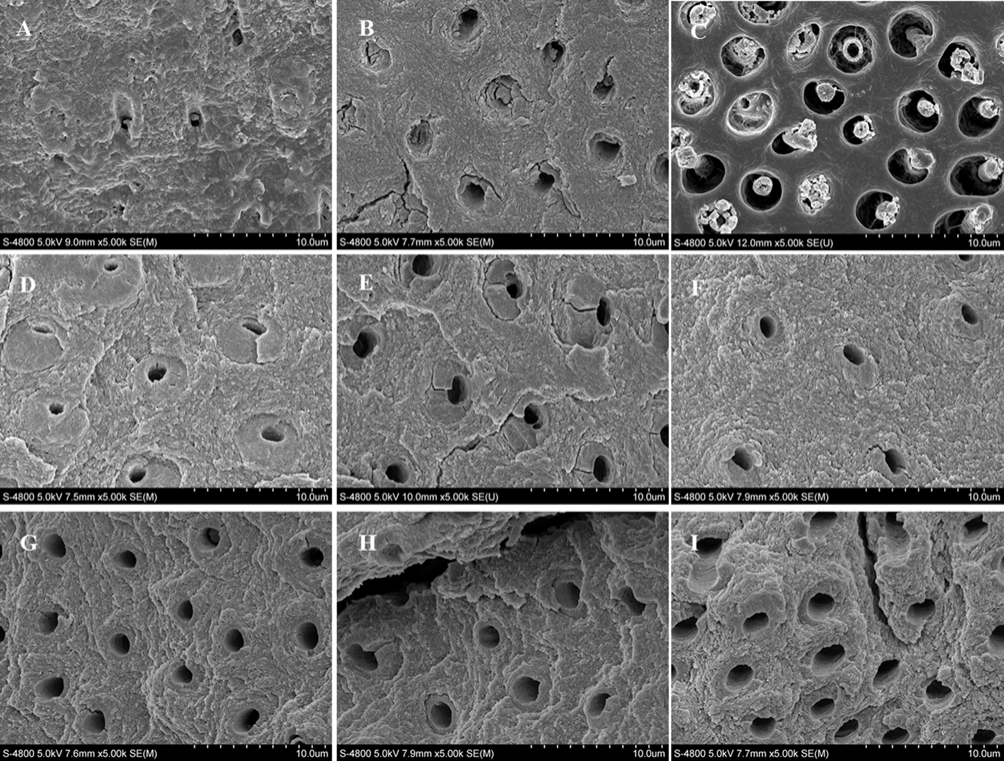
SEM micrographs of non-carious sclerotic dentin in all groups. (A) The control group without any treatment; (B) Adper Easy One adhesive treatment; (C) Diamond bur polishing followed by Adper Easy One adhesive application; (D, E, F, G, H, and I) Er,Cr:YSGG laser irradiation at 1W, 2W, 3W, 4W, 5W, and 6 W, respectively, and followed by Adper Easy One adhesive application. (magnification, 5000×).

Fig 3 shows representative SEM images of the non-carious sclerotic dentin surfaces in all groups. In the control group, the surfaces of the specimens appeared to have an amorphous structure, a contamination layer was observed on the surface of the samples, and we could observe few open dentinal tubules. In the etching group, we can see that after the use of the Adper Easy One adhesive, the superficial contamination layer disappeared. Similarly, only a few dentinal tubules remained partially exposed in SEM images, and most were filled with sclerotic casts. In the diamond bur group, most dentinal tubules were filled with mineral crystallites which partly extended outside of the opening of the dentinal tubules. In the 1W Er,Cr:YSGG laser group, we observed that the whole surfaces of non-carious sclerotic dentin appeared rougher than the above three groups, and most dentinal tubules showed open partially. In the 2W Er,Cr:YSGG laser group, the surfaces of the specimens also showed rough, furthermore, the degree of the dentinal tubules looks higher than the 1W laser group. Likewise, in the 3W, 4W, 5W and 6W Er,Cr:YSGG laser group, we can see the surfaces of non-carious sclerotic dentin in these four groups showed rough, and the dentinal tubules are all opened partially to some extent. In addition, in the 5W and 6W group, cracks appeared in the surfaces of sclerotic dentin.

**Fig 4:**
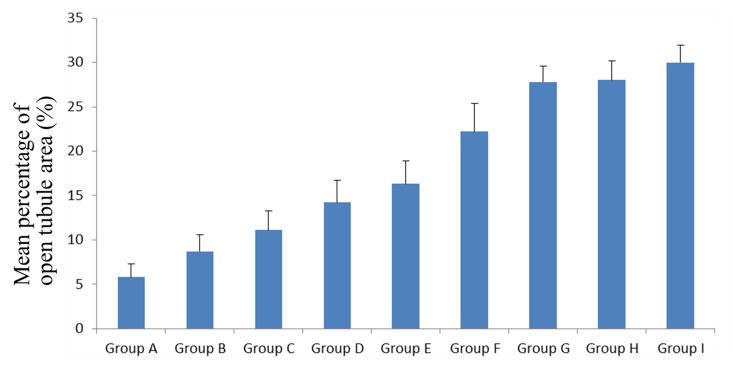
Mean percentages of open tubule areas of non-carious sclerotic dentin in all groups. (A) The control group without any treatment; (B) Adper Easy One adhesive treatment; (C) Diamond bur polishing followed by Adper Easy One adhesive application. (D, E, F, G, H, and I) Er,Cr:YSGG laser irradiation at 1W, 2W, 3W, 4W, 5W, and 6 W, respectively, and followed by Adper Easy One adhesive application.

Fig 4 provides the relative percentages of open tubule areas of non-carious sclerotic dentin in all groups. We could know by the statistical analysis the lowest mean percentage of open tubule area was observed in the control group among all the groups, however, there was no significant difference between the control group and the etching group (p>0.05). All the specimens irradiated with the Er,Cr:YSGG laser had a higher mean percentage of open tubules area than those without laser irradiation. The highest value was observed in the 6W laser group among all groups, but this value was not significantly different with those of the 4W and 5W laser group (p>0.05). Comparing the results of the etching group and the diamond bur group, we can see that the two groups yielded similar mean open tubule areas (p>0.05). Moreover, the relative percentage of open tubule area in the etching group was significantly lower than the six Er,Cr:YSGG laser groups (p<0.05), meanwhile, the result in the diamond bur group showed no statistical difference compared with the 1W laser group (p>0.05), but was significantly lower than the 2W, 3W, 4W, 5W and 6W laser group (p<0.05). From the whole figure, we can see that the relative percentage of area occupied by open tubules gradually increased from the control group to the 6W laser group.

### 2. Micro-shear bond strength

Table 2 shows the micro-shear bond strength between the resin composites and non-carious sclerotic dentin in all groups before and after aging. Two-way ANOVA indicated that both the different treatments and aging could significantly influence the micro-shear bond strengths of non-carious sclerotic dentin to resin composites (p<0.05). In the immediately tested subgroups, the micro-shear bond strength of 4W Er,Cr:YSGG laser was significantly higher than other subgroups (p<0.05), and the control subgroup showed the lowest strength in all subgroups, however, the control subgroup not statistically different with the 1W laser subgroup and 6W laser subgroup (p>0.05). The micro-shear bond strength of the diamond bur subgroup was significantly higher than the control subgroup (p<0.05) while lower than the 4W Er,Cr:YSGG laser (p<0.05). Furthermore, the bond strength of the damond bur subgroup was not significantly different with 1W, 2W, 3W, 5W and 6W laser subgroups (p>0.05), and there is no significant difference among the latter 5 laser subgroups. In the thermocycling subgroups, 4W Er,Cr:YSGG laser subgroup obtained the highest micro-shear bond strength (p<0.05), and the control subgroup was statistically lower than any other subgroup (p<0.05). There was no statistical difference among the diamond bur subgroup, 1W, 2W, 3W, 5W and 6W laser subgroup (p>0.05).

**Table 2:** Micro-shear bond strength to non-carious sclerotic dentin in different groups (Mpa).

Groups	Micro-shear bond strength	
	Immediately tested	After thermal cycling
**Control**	11.79±1.90^aA^	7.44±1.63^aB^
**Diamond bur**	14.28±1.93^bA^	11.44±2.28^bB^
**1W Er,Cr:YSGG laser**	13.15±2.09^abA^	10.12±2.31^bB^
**2W Er,Cr:YSGG laser**	13.64±2.07^bA^	11.38±2.53^bB^
**3W Er,Cr:YSGG laser**	14.49±2.13^bA^	11.85±1.98^bB^
**4W Er,Cr:YSGG laser**	17.60±2.55^cA^	14.35±2.08^cB^
**5W Er,Cr:YSGG laser**	13.79±1.95^bA^	11.22±2.29^bB^
**6W Er,Cr:YSGG laser**	13.46±1.76^abA^	10.59±2.55^bB^

Different lowercase letters indicate statistically significant differences among groups in every column (p < 0.05).

Different capital letters indicate statistically significant differences between groups in every row (p < 0.05).

In every group, the bond strength of the subgroup after thermocycling was significantly lower than the subgroup in immediately test (p<0.05).

## Discussion

We chose to use bovine teeth in the present study due to the difficulty associated with obtaining human teeth with sclerotic features. Previous studies have indicated that human and bovine dentin substrates had a similar morphology [24, 33], and this superficial similarity rationalized the use of bovine sclerotic dentin as a replacement for human sclerotic dentin in studies evaluating the performance of adhesive systems [34–36].

In the present study, the effect of different treatments on the bond property was investigated. The results revealed the group with 4W Er,Cr:YSGG laser treatment showed the optimal surface micromorphology and the bond strength compared with other groups. Thus, the null hypothesis that different treatments had no effect on the micromorphology and micro-shear bond strength of non-carious sclerotic dentin to resin composites has to be rejected.

Measurement of bond strength, regardless of the technique chosen is a controversial topic in dental adhesion [37]. In recent times researchers have preferred to use micro- tensile method and fracture mechanics to evaluate the properties of the adhesive interface of dentin [38]. However, in the present study the micro-shear bond test was adopted to measure bond strength because: (1) the non-carious sclerotic dentin of the bovines we collected was located in the lingual surface, which was difficult to make the micro-tensile test specimen. (2) in the environment of oral cavity, especially for the tooth cervical, where it usually suffers from shear force instead of merely tensile force. Therefore, the micro-shear test can simulate the clinical failure scenario more realistically than the micro-tensile test.

Compared with immediate bond strength, the durability of the bonded interface between dentin and restorations has always been a great challenge for clinicians. In vitro studies, thermocycling and thermomechanical loading are usually used to simulate the clinical conditions. Although, thermocycling can mimic thermal changes in wet oral environments, it neglects masticatory functions. Compared with thermocycling, thermomechanical loading is an optimal aging manner. However, due to the sclerosis location of dentin and the specificity of the specimen, only thermocycling can be applied.

Currently, to treat a wedge-shaped defect, dentists usually perform diamond bur polishing and apply a self-etching adhesive or simply apply the adhesive alone. Previous studies have demonstrated that although the superficial hypermineralized layer can be removed by combined treatment with diamond bur polishing and self-etching adhesive application, the treated surface will obtain a smear layer that cannot be removed with the common luting cements. In addition, the mineral crystals are still retained in the dentinal tubules [2]. Use of the adhesive only achieves results similar to those obtained with both diamond bur polishing and adhesive application [1]. Irradiation with an Er,Cr:YSGG laser, as a new hydrokinetic biological laser system, does not result in the formation of a smear layer or heat injury when it is used to pretreat the dental hard tissue as well as the pulp at appropriate parameters [16, 17]. Therefore, considering the structure characteristics of non-carious sclerotic dentin, irradiation with an Er,Cr:YSGG laser maybe effectively improve its bonding property.

The two main factors that influence bonding strength between dentin and composite resins are: the hybrid layer and resin tags. However, for non-carious sclerotic dentin, when mineralized degeneration occurs in the organic collagen, leading to replacement of the collagen-rich intertubular dentin with highly mineralized peritubular dentin, the formed hybrid layer becomes thinner and even disappears in places 3–5]. Based on previous studies, we know that compared with the hybrid layer, the surface roughness of non-carious sclerotic dentin showed more importance in bond to resin composites, just as for the enamel. Moreover, blockage of the dentinal tubules reduces the amount of potential resin tags [1, 2].

Previous studies have indicated that laser irradiation influences the bond of dental resin to enamel and dentin [36, 39], and the most important parameter controlling the effect of laser irradiation is the output power of the laser. Lower power laser irradiation results in less change to the tooth surface. Nevertheless, Visuri et al [36] showed that application of excessively high laser power can result in charring and cracking in the tooth surface, decreasing the strength of the dentin. In the present study, we observed by FESEM that on the surface of non-carious sclerotic dentin treated by the Er,Cr:YSGG laser with various powers, no smear layer was formed, and the surfaces were clean and rough. With the increase of laser power, the relative percentages of open tubule areas of the sclerotic dentin treated with the Er,Cr:YSGG laser at the higher output powers were apparently larger than those obtained in the lower laser power groups. Based on these results, we conclude that compared to the conventional treatment methods used for non-carious sclerotic dentin, Er,Cr:YSGG laser irradiation can more effectively decrease the blockage of dentinal tubules by mineralized crystals. Furthermore, surfaces treated with 5 and 6 W laser power showed cracks in FESEM images. Overall, the results of the present study are in agreement with those of previous studies [36].

Hossain et al [6, 7] found that by roughening the dentin surface, the bonding area between substrates and resin composite can be increased markedly, which is beneficial to the bonding strength. Also, in the present study, through quantification achieved using a 3D morphology scanner, we observed that the roughness values were approximately equal between some laser-treated groups and significantly higher than those achieved with the traditional pretreatment methods. Interestingly, among the groups in which samples were irradiated with the Er,Cr:YSGG laser, in the lower range of output power (0–4 W), surface roughness increased with the increase of laser power. However, in the higher output power range (>4 W), surface roughness decreased with the increase of laser power. This appears to be because irradiation with a laser power greater than 4 W could produce the superheated and bead the surface structure of non-carious sclerotic dentin, which is not beneficial to the roughness of non-carious sclerotic dentin, just like the enamel [40].

With the limitations of the present in vitro study, it may be concluded that for Er,Cr:YSGG laser treatment of non-carious sclerotic dentin, a laser power of 4 W is found to be the optimal power to improve the micromorphology and the bond strength of non-carious sclerotic dentin to resin composites. Thus, the Er,Cr:YSGG laser at 4W power can be a more effective manner to improve the bonding property of non-carious sclerotic dentin to resin composites.

## Supporting Information

S1 Dataset (XLSX)
